# Population Ageing: The Need for a Care Revolution in a World 2.0

**DOI:** 10.3390/geriatrics4030047

**Published:** 2019-08-14

**Authors:** Virginia Boccardi

**Affiliations:** Section of Gerontology and Geriatrics, Santa Maria della Misericordia Hospital, 60132 Perugia, Italy; virginia.boccardi@unipg.it; Tel.: +39-0755783524

**Keywords:** ageing, challenge, disease, frailty, care

In recent years, we have seen a substantial improvement in social and health conditions, with a significant increase in the human survival rate as never seen before. As well, there have been significant increases in the “oldest old”– the population above the age of 85 years old. Italy, after Hong Kong and Japan, is the longest-lived country in the world. According to the latest World Health Organization (WHO) data published in 2018, life expectancy in Italy is 80.5 for males and 84.9 for females, with a total life expectancy of 82.9 [1]. Population ageing is undoubtedly a demographic success, driven by many changes in society. But while life expectancy is still rising, health span defined as the years of life lived in good health is on a fixed point from years [2]. In fact, if on the one hand, the population has become more long-lived, on the other, ageing has promoted to a progressive high prevalence of chronic age-related conditions and thus years lived in disability [3]. When we think of long-lived people, the image of a very old lonely woman, living alone, mostly disabled, and affected by multiple diseases, comes to mind. Diseases that are common to older people include type 2 diabetes, chronic heart failure, cardiovascular diseases, and dementia. Although chronic diseases are increasing, both public and private health systems are in trouble when they have to manage these new needs. This is particularly evident in caring for people with dementia, a disease that has become a leading cause of the increasing burden for society, family, and old age subjects themselves. The model of the sick individual that most distinguishes modern society is not so much the individual affected by a specific disease, acute, and curable in the short to medium term, but rather a chronic and complex patient, mostly suffering from different diseases at the same time. The simultaneous presence of multiple diseases is what really characterizes geriatrics today. This also leads to the polypharmacy and a major risk in old age subjects for an adverse reaction to drugs. This condition can be associated with both the risk of fragmented interventions, focused more on the treatment of the single disease. Meeting the challenge of the ageing population and responding to the needs of older persons require a better understanding of ageing, frailty, disability, and appropriate preventive interventions as well. However, while the demand for doctors specialized in the medical care of very old persons is increasing, the interest among medical students for a career in geriatrics is lagging behind [4]. Geriatrics has characteristics that the majority of students at the beginning of their career do not find attractive. These include managing with chronically and instable ill patients, working in long-term care, less curable diseases, and dealing with social problems and end life issues. Medical students and residents found caring for the old age person unattractive because they do not know how to deal with the complexity involved, multimorbidity, polypharmacy, shorter life expectancy, and balancing treatment of diseases with quality-of-life and psychosocial issues [5]. Yet it is precisely these characteristics that have made me love geriatrics. Because geriatrics also has another face. The oldest represent the greatest example of adaptation, despite all the physical and psycho-affective changes to which he is subjected. Retirement with loss of an active profession or societal role, fewer opportunities for social interaction, due to the loss of peers or their relatives, very often give rise to a situation of solitude or real social isolation. The disease burden can, more easily than in other ages, help to determine or increase individual insecurity or affect its self-esteem by encouraging anxiety or/and depression, the latter being an extremely common condition in old age. However, the older adapts and survives such radical changes. Today, thanks to the general well-being, a large part of the older population live at the height of its strength, often well integrated into the daily reality of work and family, so much so that its experience can be of fundamental support for the development of society. Faced with a possible reduction in the near future in the number of frail older subjects thanks to greater prevention and culture on the psychophysical well-being that must lead to a more advanced age, we cannot fail to underline the stringent need to improve knowledge and the approach to fragility to ensure a dignified life, even in the presence of disability and chronicity, with highly personalized care and therapeutic interventions. In modern society 2.0, a strong emphasis on prevention of complications related to chronic diseases and the promotion of healthy and active ageing are needed in order to delay the onset of disability and dependency. The main goal for geriatricians is to follow patients for any changes in function and trying to improve their abilities even though diseases are not curable. Again, geriatricians should focus specifically on those conditions that can affect a person’s functional abilities and quality of life. Appropriate services for older persons, when they do develop disabilities, are necessary for the community and hospitals, considering that these latter are constructed and conceived for younger, and not for old age subjects and their needs. Such a heterogeneous population requires intensive management in acute setting, whereas their treatment and subsequent discharge can prove many challenging. To this aim, space and services tailored to older are strongly needed. The intermediate care requires improvement both to prevent inappropriate hospitalization as well as to help and facilitate geriatricians with difficult discharges. Also the last phase of people’s lives challenges the traditional care attitude. Geriatric palliative care should be conceptualized as an interdisciplinary field of care based on the synergies between geriatric medicine and palliative care. It is central to promote geriatric culture among different professional figures to deliver care that responds to the needs and expectations of the patients and their families. The severe ill oldest persons constitute a vulnerable population and their wellbeing depend on “care” that must be multidimensional and oriented towards a person’s respect avoiding obstinacy. Indeed, transferring funds and resources into primary and community-based care, supporting family caregiving, and enhancing access to health care can also contribute to meeting the challenges of an ageing population.
